# The identification and epidemiology of meticillin-resistant *Staphylococcus aureus* and *Clostridium difficile* in patient rooms and the ward environment

**DOI:** 10.1186/1471-2334-13-342

**Published:** 2013-07-24

**Authors:** Meredith C Faires, David L Pearl, Olaf Berke, Richard J Reid-Smith, J Scott Weese

**Affiliations:** 1 Department of Population Medicine, University of Guelph, Guelph, ON, Canada; 2 Department of Mathematics and Statistics, University of Guelph, Guelph, ON, Canada; 3 Department of Pathobiology, University of Guelph, Guelph, ON, Canada

**Keywords:** Meticillin-resistant *Staphylococcus aureus*, *Clostridium difficile*, Epidemiology, Environment, Hospital, Ward, Patient rooms, Risk factors, Ribotype 078

## Abstract

**Background:**

Research has indicated that the environment may play an important role in the transmission of meticillin-resistant *Staphylococcus aureus* (MRSA) and *Clostridium difficile* in healthcare facilities. Despite the significance of this finding, few data exist from longitudinal studies investigating MRSA and *C. difficile* contamination, concurrently, in both patient rooms and the general ward environment. The objectives of this study were to determine the prevalence of MRSA and *C. difficile* contamination in patient rooms and the ward environment and identify risk factors associated with a surface being contaminated with these pathogens.

**Methods:**

Environmental surfaces in patient rooms and the general environment in the medical and surgical wards of a community hospital were sampled six times over a 15 week period. Sterile electrostatic cloths were used for sampling and information pertaining to the surface sampled was recorded. MRSA isolates and *C. difficile* specimens were obtained from hospitalized patients.

Enrichment culture was performed and *spa* typing or ribotyping was conducted for MRSA or *C. difficile*, respectively. Exact logistic regression models were constructed to examine risk factors associated with MRSA and *C. difficile* contamination.

**Results:**

Sixteen (41%) patient rooms had ≥ 1 surfaces contaminated with MRSA and/or *C. difficile*. For 218 surfaces investigated, 3.2% and 6.4% were contaminated with MRSA or *C. difficile*, respectively. Regression models indicated that surfaces in rooms exposed to a *C. difficile* patient had significantly increased odds of being contaminated with *C. difficile*, compared to surfaces in unexposed patient rooms. Additionally, compared to plastic surfaces, cork surfaces had significantly increased odds of being contaminated with *C. difficile*. For 236 samples collected from the ward environment, MRSA and *C. difficile* were recovered from 2.5% and 5.9% of samples, respectively. Overall, the majority of MRSA and *C. difficile* strains were molecularly identified as *spa* type 2/t002 (84.6%, n = 11) and ribotype 078 (50%, n = 14), respectively.

**Conclusions:**

In patient rooms and the ward environment, specific materials and locations were identified as being contaminated with MRSA or *C. difficile*. These sites should be cleaned and disinfected with increased vigilance to help limit the transmission and dissemination of MRSA and *C. difficile* within the hospital.

## Background

In Canadian hospitals, the incidence of meticillin-resistant *Staphylococcus aureus* (MRSA) infections and colonization have increased 17-fold from 1995 to 2007 [1] and remains a leading cause for a range of opportunistic infections [1]. Similarly, *Clostridium difficile* infection (CDI) has also increased in incidence and severity including a four-fold increase in attributed mortality [2], and is the most commonly diagnosed cause of healthcare-associated (HA) and antimicrobial-associated diarrhea [3].

Patients are the main reservoirs of both these pathogens, but there is increasing evidence that the environment may play a significant role in the nosocomial transmission of MRSA [4] and *C. difficile*[5]. The ability of contaminated surfaces contributing to the transmission of nosocomial pathogens relies on several determinants including the frequency at which surfaces are contaminated, if levels of contamination are sufficiently high to result in transmission, and the ability of pathogens to remain viable on various surfaces [6]. Research has demonstrated that *S. aureus*[7] and *C. difficile* spores [8] are able to survive and persist in the hospital environment for months. As levels of environmental contamination with these HA pathogens increase, the prevalence of healthcare worker hand carriage also increases [9,10]. Additionally, patients and visitors may be contaminated from contaminated surfaces, creating further potential for acquisition or transmission of MRSA and *C. difficile* in the healthcare setting and the community.

Studies investigating contamination of hospital surfaces have primarily focused on MRSA or *C. difficile*. There has been limited research investigating environmental contamination with both of these pathogens, concurrently, in healthcare facilities. Furthermore, potential associations or commonalities between environmental contamination with MRSA and *C. difficile*, in both patient rooms and the ward environment have not been explored. Given the different sources of MRSA and *C. difficile* contamination, nasal/wound versus fecal, and different susceptibility to disinfectants, an understanding of the epidemiology of MRSA and *C. difficile* in the environment, in parallel study, can help infer sources of contamination and potential factors associated with contamination. Subsequently, the data obtained from this research can be used by hospital personnel to evaluate hospital infection protocols and procedures.

The objectives of this investigation were to determine the prevalence and identify risk factors associated with MRSA and *C. difficile* contamination in patient rooms and the general ward environment of a community hospital and compare MRSA and *C. difficile* strains between patients and the contaminated environment.

## Methods

### Study site

The participating healthcare facility is a 260-bed large community hospital located in Ontario, Canada. Starting in December 2010, environmental sampling of patient rooms and the general hospital environment in the medical and surgical wards was conducted during six visits over a 15 week period. Sampling was conducted once a week for three consecutive weeks during weeks 1–3 (visits 1–3) and weeks 13–15 (visits 4–6). During the study period, the MRSA colonization and infection rate ranged from 0.38 – 1.93 cases/1,000 patient days and the CDI rate ranged from 0.19 – 0.83 cases/1,000 patient days. Although a *C. difficile* outbreak did not occur during the study period, the hospital did experience an MRSA outbreak between weeks 8 and 13 and an influenza outbreak between weeks 9 and 11. However, both outbreaks occurred in wards not involved in this investigation. For environmental sampling in patient rooms, verbal consent from patients, or from a parent in the case of a child, was required prior to sampling. Written informed consent was not required as surfaces sampled in patient rooms belonged to the hospital and sampling of patient’s personal belongings was not conducted. The University of Guelph and the hospital research ethics boards approved this study.

### Sampling

Sampling of environmental surfaces was conducted in patient rooms and the ward environment of one surgical ward and two medicine wards, identified as medicine A and medicine B. Three different types of patient rooms were sampled for contamination. An unexposed room had a patient not identified with MRSA or CDI and was not under isolation precautions. A *C. difficile* exposed room had a patient identified with CDI, based on clinical signs and a positive stool toxin ELISA test (Tox A/B Quik Chek, TechLab, Blacksburg, Virginia, USA), and was under isolation precautions. An MRSA exposed room had a patient identified with an MRSA infection and/or colonization, based on microbiological diagnosis, and was under isolation precautions. In the participating hospital, surveillance for MRSA is conducted based on recommendations provided by a Provincial Infectious Disease Advisory Committee [11]. Briefly, at the time of hospital admission, patients identified as having an increased risk for MRSA acquisition (e.g., specific risk factors, wounds, incisions, catheter sites) are screened. For patients located on high risk units (e.g., intensive care unit), universal admission screening is performed. During hospitalization, surveillance cultures are not routinely conducted. Furthermore, screening of catheter sites are performed only if clinical signs of infection are observed or the patient is transferred to the continuing care or rehabilitation wards.

For each MRSA and *C. difficile* exposed room that was identified, two unexposed rooms were randomly sampled on the same ward as the exposed room. In all patient rooms, six different surfaces were sampled including the railing at the end of the bed, bulletin board, chair back, overbed table, privacy curtain, and television. For unexposed rooms, if more than one patient was present, the investigator randomly chose a patient and sampled the surfaces in that patient’s vicinity. For the general ward environment, environmental surfaces sampled were those distributed over the ward and located in nursing and physician work areas, hallways, and visiting rooms. Surfaces were sampled 4–6 times over the study period and prior to being cleaned by housekeeping staff. In the participating facility, surfaces located in the general ward environment and patient rooms (excluding *C. difficile* exposed rooms*)*, were cleaned once a day using a quaternary ammonium compound. In *C. difficile* exposed rooms*,* surfaces were cleaned twice a day with bleach. Following discharge, all patient rooms were subjected to a terminal clean with a quaternary ammonium compound. In addition, *C. difficile* exposed rooms were cleaned with bleach. No changes in the hospital cleaning protocol on the surgical and medicine wards were instituted during the study period.

Sterile electrostatic cloths (Swiffer™, Proctor and Gamble, USA) were used for environmental sampling. Using a gloved hand, the investigator wiped the electrostatic cloth over half the environmental surface to be sampled, up to a maximum of 20 cm × 20 cm. The cloth was then placed in a sterile collection bag (Whirl-Pak®, Nasco, California, USA). The investigator then used a second cloth to sample the other half of the environmental surface. Gloves were changed between each sampling. During each visit, two electrostatic cloths were not used for sampling but were handled and processed in parallel, to act as negative controls. Information collected with each surface sampled included: visit number, hospital ward, surface location, environmental surface sampled, and type of surface material. From the hospital’s microbiology laboratory, patient isolates identified as MRSA were collected from culture plates using a culture swab with Stuart’s media and stool samples from *C. difficile* patients were also obtained. Isolates or specimens were obtained from only patients that were hospitalized in the medicine and surgical wards during the 15 week study period, irrespective of whether they were present in the wards on the sampling date. Only one isolate per patient was analyzed. Isolates from patients that were epidemiologically linked to the MRSA outbreak were not collected. All specimens submitted for MRSA or *C. difficile* testing were collected at the discretion of medical personnel.

### Processing

For MRSA, cloths were immersed in 30 ml of enrichment broth containing sodium chloride (75 g/L), mannitol (10 g/L), tryptone (10 g/L), and yeast extract (2.5 g/L) and incubated at 35°C, aerobically, for 24 hours. Following incubation, a 5 μL aliquot of broth was inoculated onto MRSA Chromogenic agar (BBL CHROMagar MRSA, Becton, Dickinson and Company, Sparks, Maryland, USA) and incubated, aerobically, at 35°C for 24–48 hours. Isolates were identified as *S. aureus* by Gram stain, tube coagulase test, catalase test, and the *S. aureus* latex agglutination assay (Pastorex Staph-plus, Bio-Rad Laboratories Ltd., Mississauga, Ontario, Canada). Meticillin-resistance was confirmed by testing for the penicillin-binding protein 2a (MRSA Screening Kit, Denka Seiken, Campbell, California, USA). For patient isolates, culture swabs were streaked onto blood agar (Oxoid, Nepean, Ontario, Canada) and processed as described above. Molecular typing of MRSA was conducted using sequence analysis of the X region of the staphylococcal protein A gene (*spa* typing) [12]. Sequences were analyzed using the eGenomics software [13] and the Ridom system [14]. For eGenomics, *spa* types are reported using a numerical system (e.g., *spa* type 2) whereas Ridom *spa* types are reported using a numerical system preceded by a ‘t’ (e.g., t002). All *spa* types were compared to epidemic MRSA strains that are commonly found in Canada [15]. These Canadian epidemic MRSA strains are designated as CMRSA and should not be confused with community-associated MRSA strains which are designated as CA-MRSA. All MRSA isolates were investigated for the *luk*F-PV gene encoding the Panton-Valentine leukocidin toxin by real-time PCR [16]. Molecular typing of the MRSA outbreak strain was not conducted by the hospital.

For *C. difficile*, electrostatic cloths were immersed in 30ml of enrichment broth containing brain-heart infusion (37 g/L) and 0.1% sodium taurocholate and incubated at 37°C, anaerobically, for 5 days. A 2 ml sample of broth was then alcohol shocked for one hour, followed by centrifugation at 4,000 rpm for 10 minutes. The resulting pellet was inoculated onto *C. difficile* moxalactam-norfloxacin agar (Oxoid, Nepean, Ontario, Canada) and incubated for 24–96 hours at 37°C, anaerobically. Colonies were confirmed as *C. difficile* based on characteristic morphology, odour, and production of L-proline-aminopeptidase (Prodisk, Remel, Lenexa, Kansas, USA). For patient isolates, approximately 1 g of feces was inoculated into 9 ml of enrichment broth and processed as described previously. All isolates identified as *C. difficile* were investigated for the presence of toxin A (*tcdA*) [17], toxin B (*tcdB*) [18], and the binary toxin (*cdtA*) [19] genes using PCR. Ribotyping was used to analyze all *C. difficile* isolates [20]. When a ribotype pattern was identified as an international ribotype based on comparison to reference strains, the appropriate numerical designation (e.g., 027) was assigned. Alternatively, an internal laboratory designation was assigned. Toxinotyping [21] was also performed on a representative of each toxigenic ribotype.

### Statistical analysis

For patient rooms and the general ward environment, the prevalence of MRSA and *C. difficile* contamination was determined for ward, location, surface material, surface sampled, and visit (ward only). Statistical models were also constructed, using exact logistic regression, to test for associations between the occurrence of environmental contamination with MRSA or *C. difficile* and the following predictor variables: ward, location, surface material, surface sampled, and visit number (ward only). Additionally, the overall prevalence of surfaces positive for MRSA or *C. difficile* prior to (visits 1–3), and following (visits 4–6), the MRSA and influenza outbreaks, were compared using exact logistic regression. The Spearman’s rank correlation test was used to identify correlations between all independent variables. Variables with a correlation of >0.8 were investigated and only the variable that was more biologically plausible was included in the model to avoid issues associated with collinearity [22]. Due to the relatively low prevalence of MRSA and *C. difficile* contamination in both patient rooms and the ward environment, only univariable models were constructed using exact logistic regression, employing the score method to calculate the P-value [23]. Compared to other computational methods, the score method does not compute an equivalent confidence interval; subsequently, only odds ratios (OR) and P-values are presented. For significant independent variables with several categories, contrasts between categories were also examined. Descriptive statistics and analyses were performed using Stata 11.0 (StataCorp LP, College Station, Texas, USA). All tests were two-sided and the significance level was selected at α = 0.05.

## Results

### Patient rooms

A total of 218 surfaces located in 26 unexposed rooms, 10 MRSA exposed rooms, and 3 *C. difficile* exposed rooms, were sampled during the study period. The prevalence of MRSA and *C. difficile* contamination in each type of patient room by ward, surface material, and surface sampled is presented in Table 1. Overall, the highest prevalence of MRSA contamination occurred in unexposed rooms and the highest prevalence of *C. difficile* contamination occurred in *C. difficile* exposed rooms. A total of 12 (46.2%) unexposed rooms were previously occupied by a MRSA patient. However, none of the surfaces in these 12 rooms were contaminated with MRSA. During the study period, none of the unexposed rooms were previously occupied by a *C. difficile* patient.

**Table 1:** **Descriptive statistics of variables for MRSA and *C. difficile *contamination in patient rooms**

	**Unexposed rooms (n = 26)**		**MRSA exposed rooms (n = 10)**		***C. difficile *exposed rooms (n = 3)**		**Overall Prevalence**	
**Variables**^**a**^	**MRSA (%)**	***C. difficile *(%)**	**MRSA (%)**	***C. difficile *(%)**	**MRSA (%)**	***C. difficile *(%)**	**MRSA (%)**	***C. difficile *(%)**
General prevalence	6/149 (4.0)	9/149 (6.0)	1/52 (1.9)	1/52 (1.9)	0/17 (0)	4/17 (23.5)	7/218 (3.2)	14/218 (6.4)
Ward:								
Medicine A^b^	4/54 (7.4)	3/54 (5.6)	0/20 (0)	1/20 (5.0)	0/5 (0)	2/5 (40)	4/79 (5.1)	6/79 (7.6)
Medicine B^c^	0/35 (0)	3/35 (8.6)	1/16 (6.3)	0/16 (0)	No sampling	No sampling	1/51 (1.9)	3/51 (5.9)
Surgery^d^	2/60 (3.3)	3/60 (5.0)	0/16 (0)	0/16 (0)	0/12 (0)	2/12 (16.7)	2/88 (2.3)	5/88 (5.7)
Material type:								
Cork	0/23 (0)	3/23 (13)	0/7 (0)	1/7 (14.3)	0/3 (0)	2/3 (66.7)	0/33 (0)	6/33 (18.2)
Fabric	0/26 (0)	0/26 (0)	1/8 (12.5)	0/8 (0)	0/2 (0)	0/2 (0)	1/36 (2.8)	0/36 (0)
Laminate	1/26 (3.8)	1/26 (3.8)	0/9 (0)	0/9 (0)	0/3 (0)	1/3 (33.3)	1/38 (2.6)	2/38 (5.3)
Plastic	5/74 (6.8)	5/74 (6.8)	0/28 (0)	0/28 (0)	0/9 (0)	1/9 (11.1)	5/111 (4.5)	6/111 (5.4)
Surface sampled:								
Bulletin board^e^	0/23 (0)	3/23 (13)	0/7 (0)	1/7 (14.3)	0/3 (0)	2/3 (66.7)	0/33 (0)	6/33 (18.2)
Chair back^e^	4/25 (16)	2/25 (8)	0/10 (0)	0/10 (0)	0/3 (0)	0/3 (0)	4/38 (10.5)	2/38 (5.3)
End of bed	1/26 (3.8)	1/26 (3.8)	0/10 (0)	0/10 (0)	0/3 (0)	1/3 (33.3)	1/39 (2.6)	2/39 (5.1)
Overbed table^e^	1/26 (3.8)	1/26 (3.8)	0/9 (0)	0/9 (0)	0/3 (0)	1/3 (33.3)	1/38 (2.6)	2/38 (5.3)
Privacy curtain^e^	0/26 (0)	0/26 (0)	1/8 (12.5)	0/8 (0)	0/2 (0)	0/2 (0)	1/36 (2.8)	0/36 (0)
Television^e^	0/23 (0)	2/23 (8.7)	0/8 (0)	0/8 (0)	0/3 (0)	0/3 (0)	0/34 (0)	2/34 (5.9)

n = number of rooms.

^a^ Denominator is based on the number of surfaces in patient rooms that were tested.

^b^ Total of 10 unexposed rooms, 4 MRSA exposed rooms, 1 *C. difficile* exposed room located in ward Medicine A.

^c^ Total of 6 unexposed rooms, 3 MRSA exposed rooms, 0 *C. difficile* exposed rooms located in ward Medicine B.

^d^ Total of 10 unexposed rooms, 3 MRSA exposed rooms, 2 *C. difficile* exposed rooms located in the surgical ward.

^e^ Surfaces were not present in all patient rooms at the time of sampling.

Seven MRSA isolates were recovered from patient rooms in which two different *spa* types were identified; both consistent with the CMRSA-2 strain (Table 2). For *C. difficile*, four different ribotypes were identified among the 14 isolates recovered, including internationally recognized ribotypes 027 (n = 1) and 078 (n = 9) (Table 3).

**Table 2:** Typing data for MRSA strains from patients and contaminated surfaces

***spa *type**^**a**^	**Number of isolates**	**Ridom *spa *type**^**b**^	**PVL gene**	**CMRSA**	**USA equivalent**
Patient rooms:	(n = 7)				
2^c^	85.7% (6)	t002	No	2	100
437^d^	14.3% (1)	t003	No	2	100
General environment:	(n = 6)				
2	83.3% (5)	t002	No	2	100
1	16.7% (1)	t008	Yes	10	300
Patients^e^:	(n = 46)				
2^f^	65.2% (30)	t002	No	2	100
437	13% (6)	t003	No	2	100
New^g^	6.5% (3)	Not assigned	No	Not assigned	Not assigned
1	4.3% (2)	t008	Yes	10	300
7	2.2% (1)	Not assigned	No	Not assigned	Not assigned
12	2.2% (1)	t062	No	2	100
23	2.2% (1)	t548	No	2	100
771^h^	2.2% (1)	t2104	Yes	Not assigned	Not assigned
New	2.2% (1)	t3618	No	Not assigned	Not assigned

n = number of isolates.

PVL = Panton-Valentine leukocidin.

CMRSA = Canadian epidemic meticillin-resistant *Staphylococcus aureus.*

^a^*spa* types classified according to eGenomics (http://tools.egenomics.com).

^b^*spa* types classified according to the Ridom system (http://www.spaserver.ridom.de).

^c^ Five isolates recovered from five unexposed rooms; one isolate recovered from one MRSA exposed room.

^d^ One isolate recovered from one unexposed room.

^e^ For three MRSA exposed rooms, the patient’s MRSA isolate was not collected.

^f^ Five MRSA isolates are from MRSA exposed rooms that were sampled.

^g^ One patient isolate from a MRSA exposed room.

^h^ Patient isolate from a MRSA exposed room.

**Table 3:** **Typing data for *C. difficile *strains from patients and contaminated surfaces**

**Ribotype**	**Number of isolates**	**Toxinotype**	**Toxin genes**
Patient rooms:	(n = 14)		
078^a^	64.3% (9)	V	*tcdA, tcdB, cdtA*
MOH-AI^b^	21.4% (3)	0	*tcdA, tcdB*
027^c^	7.1% (1)	III	*tcdA, tcdB, cdtA*
MOH-V^c^	7.1% (1)	0	*tcdA, tcdB*
General environment:	(n = 14)		
078	35.7% (5)	V	*tcdA, tcdB, cdtA*
027	14.3% (2)	III	*tcdA, tcdB, cdtA*
MOH-V	14.3% (2)	0	*tcdA, tcdB*
001	7.1% (1)	0	*tcdA, tcdB*
MOH-AG	7.1% (1)	0	*tcdA, tcdB*
MOH-T	7.1% (1)	0	*tcdA, tcdB*
MOH-U	7.1% (1)	0	*tcdA, tcdB*
UNK-2	7.1% (1)	Not tested	None
Patients^d^:	(n = 21)		
027^e^	33.3% (7)	III	*tcdA, tcdB, cdtA*
MOH-C	14.3% (3)	IX	*tcdA, tcdB, cdtA*
CHP-A^f^	9.5% (2)	XXIV	*tcdA, tcdB, cdtA*
MOH-AD	9.5% (2)	III	*tcdA, tcdB, cdtA*
078	4.8% (1)	V	*tcdA, tcdB, cdtA*
CHP-C	4.8% (1)	XXIV	*tcdA, tcdB, cdtA*
CHP-D	4.8% (1)	0	*tcdA, tcdB*
MOH-M	4.8% (1)	0	*tcdA, tcdB*
MOH-Q	4.8% (1)	XII	*tcdA, tcdB*
MOH-V	4.8% (1)	0	*tcdA, tcdB*
MOH-Y	4.8% (1)	III	*tcdA, tcdB, cdtA*

*n* number of isolates.

*tcdA* = toxin A.

*tcdB* = toxin B.

*cdtA* = binary toxin.

^a^ One isolate from four different unexposed rooms; one isolate from one MRSA exposed room; two isolates each from two different *C. difficile* exposed rooms.

^b^ Isolates from two different unexposed rooms.

^c^ Isolate from one unexposed room.

^d^ For one *C. difficile* exposed room, *C. difficile* could not be isolated from the patient’s specimen.

^e^ One patient isolate from a *C. difficile* exposed room.

^f^ One patient isolate from a *C. difficile* exposed room.

For six patient rooms, more than one surface was contaminated with MRSA or *C. difficile* at the time of sampling. One unexposed room had MRSA recovered on two surfaces, with both MRSA isolates identified as *spa* type 2/t002. For two unexposed rooms and two *C. difficile* exposed rooms, *C. difficile* was recovered from two surfaces at the time of sampling. In three of these patient rooms, the *C. difficile* isolates recovered from the two surfaces were molecularly indistinguishable and were identified as ribotype 078.

Results from the univariable analyses (Table 4) indicated that the odds of a surface being contaminated with *C. difficile* were significantly greater in a *C. difficile* exposed room compared to an unexposed room. In addition, in patient rooms, the odds of a cork surface being contaminated with *C. difficile* were significantly greater than surfaces covered in plastic. For the dependent variable, surfaces contaminated with *C. difficile*, statistically significant contrasts were identified between *C. difficile* exposed patient rooms versus MRSA exposed patient rooms (OR 14.88; P = 0.012) and cork surfaces versus fabric surfaces (OR 10.33; P = 0.009). For MRSA contamination of surfaces in patient rooms, no variables were identified as statistically significant.

**Table 4:** **Univariable regression analyses of variables associated with MRSA or *C. difficile *contamination in patient rooms**

**Variable**	**Description**	**OR**	**P-value**	**Overall P-value for the variable**
**Dependent variable: surface contaminated with MRSA**				
Ward	Medicine A	Referent		0.614
	Medicine B	0.38	0.648	
	Surgery	0.44	0.423	
Type of patient room	Unexposed	Referent		0.594
	MRSA exposed	0.47	0.679	
	*C. difficile* exposed	1.06^a^	0.634	
Surface material	Plastic	Referent		0.776
	Cork	0.49^a^	0.346	
	Fabric	0.61	0.999	
	Laminate	0.57	0.693	
Surface sampled	End of bed	Referent		0.111
	Bulletin board	1.18^a^	0.999	
	Chair back	4.39	0.200	
	Overbed table	1.03	0.999	
	Privacy curtain	1.08	0.999	
	Television	1.15^a^	0.999	
**Dependent variable: surface contaminated with *C. difficile***				
Ward	Medicine A	Referent		0.884
	Medicine B	0.76	0.744	
	Surgery	0.73	0.758	
Type of patient room	Unexposed	Referent		0.008
	MRSA exposed	0.31	0.301	
	*C. difficile* exposed	4.71	0.031	
Surface material	Plastic	Referent		0.017
	Cork	3.84	0.030	
	Fabric	0.36	0.336	
	Laminate	0.97	0.999	
Surface sampled	End of bed	Referent		0.059
	Bulletin board	4.03	0.131	
	Chair back	1.03	0.999	
	Overbed table	1.03	0.999	
	Privacy curtain	0.44^a^	0.494	
	Television	1.15	0.999	

OR = odds ratio.

^a^ Median unbiased estimates.

### General environment

Over the study period, 45 different environmental surfaces, for a total of 236 samples, were investigated for MRSA and *C. difficile* contamination. On average, each surface was sampled 5.2 times (range 4–6). Overall, 2.5% (n = 6) and 5.9% (n = 14) of surfaces were contaminated with MRSA and *C.* difficile, respectively. During the study period, none of the control electrostatic cloths tested positive for MRSA or *C. difficile*.

Data pertaining to the distribution of MRSA and *C. difficile* contamination per ward, surface location, surface material, and surface sampled are presented in Table 5. Overall, the highest numbers of surfaces contaminated with MRSA or *C. difficile* were located in nursing and physician work areas or the hallway. Specifically, chairs and counter tops were found to be contaminated with MRSA. For *C. difficile*, surfaces identified with the most contamination included chairs, computer keyboards, and heating oven handles. However, no surfaces had increased odds of contamination compared to other surfaces for MRSA or *C. difficile* contamination based on the univariable analyses (Table 6).

**Table 5:** **Descriptive statistics of variables for MRSA and *C. difficile *contamination in the ward environment**

**Variables**^**a**^	**MRSA**	***C. difficile***
Ward:		
Medicine A (n = 72)	5.6% (4)	8.3% (6)
Medicine B (n = 67)	1.5% (1)	4.5% (3)
Surgery (n = 97)	1.0% (1)	5.2% (5)
Surface location:		
Nurses’ and physician work areas (n = 99)	1.0% (1)	6.1% (6)
Hallway (n = 109)	3.7% (4)	5.5% (6)
Visiting room (n = 28)	3.6% (1)	7.1% (2)
Surface material:		
Fabric (n = 78)	2.6% (2)	6.4% (5)
Laminate (n = 15)	13.3% (2)	0% (0)
Plastic (n = 103)	1.9% (2)	6.8% (7)
Rubber (n = 17)	0% (0)	5.9% (1)
Wood (n = 23)	0% (0)	4.3% (1)
Surface sampled:		
Chair (n = 55)	3.6% (2)	7.3% (4)
Clean linen (n = 15)	0% (0)	6.7% (1)
Clean towels (n = 16)	0% (0)	0% (0)
Computer keyboard (n = 55)	1.8% (1)	5.5% (3)
Counter top (n = 15)	13.3% (2)	0% (0)
Drug cart (n = 17)	0% (0)	5.9% (1)
Glove box holder (n = 13)	7.7% (1)	7.7% (1)
Hand rail (n = 17)	0% (0)	5.9% (1)
Heating oven handle (n = 17)	0% (0)	17.6% (3)
Patient chart (n = 16)	0% (0)	0% (0)

n = number of samples.

^a^ Denominator is based on the number of surfaces in the general ward environment that were tested.

**Table 6:** **Univariable regression analyses of variables associated with MRSA or *C. difficile *contamination in the ward environment**

**Variable**	**Description**	**OR**	**P-value**	**Overall P-value for the variable**
**Dependent variable: surface contaminated with MRSA**				
Visit	1	Referent		0.390
	2	0.42	0.628	
	3	0.35	0.617	
	4	0.31	0.361	
	5	0.26^a^	0.242	
	6	0.37^a^	0.267	
Ward	Medicine A	Referent		0.185
	Medicine B	0.26	0.368	
	Surgery	0.18	0.165	
Surface material	Plastic	Referent		0.099
	Fabric	1.33	0.999	
	Laminate	7.53	0.078	
	Rubber	2.51^a^	0.999	
	Wood	1.85^a^	0.999	
Location	Hallway	Referent		0.492
	Visiting Room	0.97	0.999	
	Work area	0.27	0.372	
Surface sampled	Computer keyboard	Referent		0.176
	Chair	2.03	0.999	
	Clean towels	1.77	0.999	
	Counter top	7.96	0.114	
	Drug cart	3.24^a^	0.999	
	Heating oven handles	3.24^a^	0.999	
	Other^b^	1.19	0.999	
**Dependent variable: surface contaminated with *C. difficile***				
Visit	1	Referent		0.439
	2	0.31	0.380	
	3	0.52	0.677	
	4	1.25	0.999	
	5	0.25	0.361	
	6	0.35	0.404	
Ward	Medicine A	Referent		0.609
	Medicine B	0.52	0.496	
	Surgery	0.59	0.531	
Surface material	Plastic	Referent		0.936
	Fabric	0.94	0.999	
	Laminate	0.69^a^	0.593	
	Rubber	0.86	0.999	
	Wood	0.63	0.999	
Location	Hallway	Referent		0.999
	Visiting Room	1.32	0.999	
	Work area	1.11	0.999	
Surface sampled	Computer keyboard	Referent		0.434
	Chair	1.36	0.999	
	Clean towels	0.58	0.999	
	Counter top	0.93^a^	0.593	
	Drug cart	1.08	0.999	
	Heating oven handles	3.63	0.139	
	Other^b^	0.79	0.999	

^a^ Median unbiased estimates.

^b^ Clean linen, glove box holder, hand rail, patient charts.

**Figure 1:**
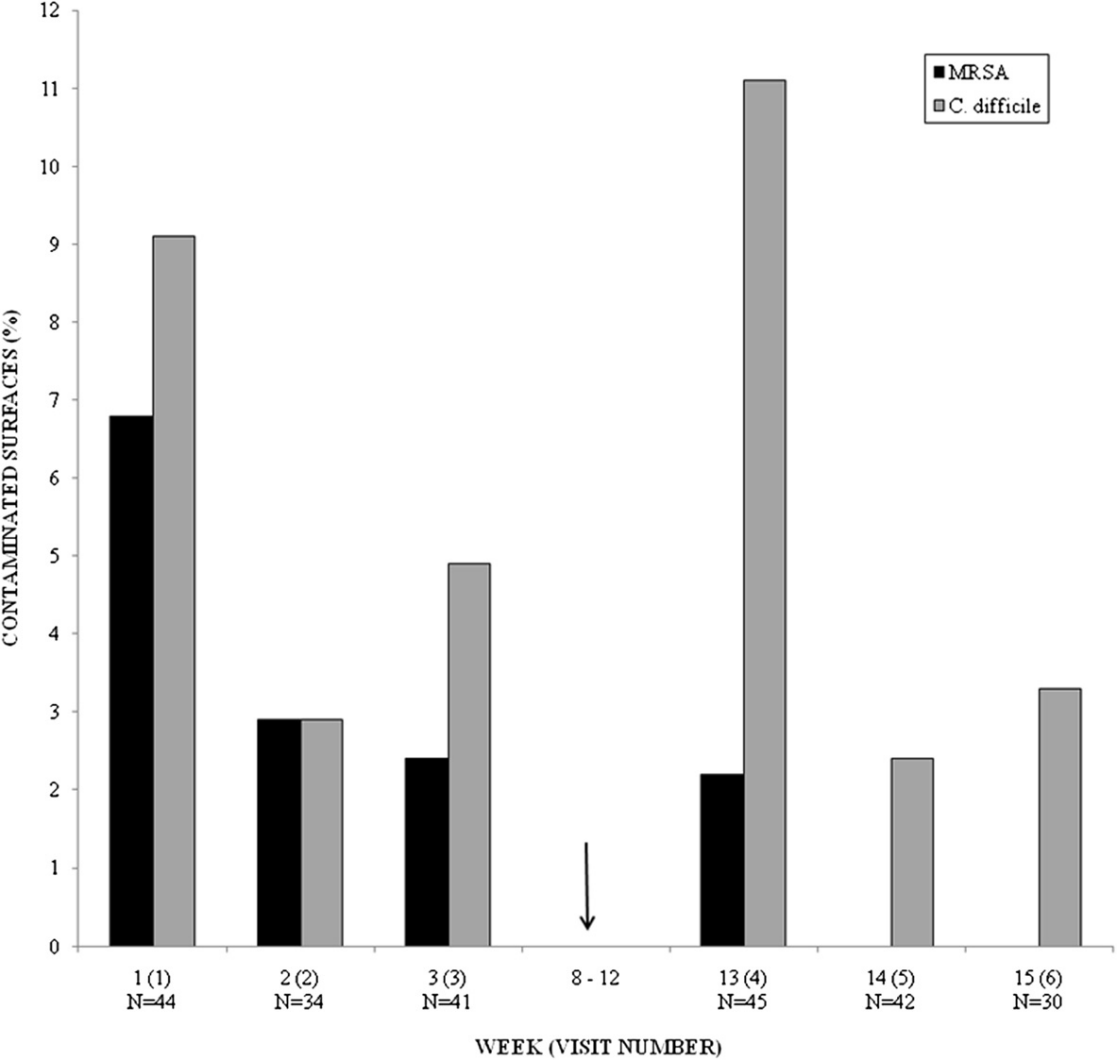
**Distribution of MRSA and *C. difficile *contamination in the ward environment by week and visit.** The arrow indicates when an MRSA outbreak (weeks 8–13) and an influenza outbreak (weeks 9–11) occurred in the hospital. N = number of surfaces sampled.

The distribution of MRSA and *C. difficile* contamination, per visit, fluctuated during the study period (Figure 1). For visits 5 and 6, MRSA was not recovered from the general ward environment. The highest prevalence of *C. difficile* contamination occurred during visit 4. Compared to the time period prior to the hospital outbreaks, there was no statistically significant increase in the odds of contamination with MRSA (OR 0.19; P = 0.213) or *C. difficile* (OR 1.02; P = 0.999) in the time period following the outbreaks.

Two different *spa* types were identified among the six MRSA isolates collected from the general ward environment (Table 2). One isolate was identified containing the PVL toxin gene and corresponded to CMRSA-10. For *C. difficile*, eight different ribotypes were identified among the 14 isolates (Table 3), including ribotypes 027 (n = 2) and 078 (n = 5).

Only one surface was positive for MRSA and *C. difficile* on the same visit; a chair located in a nursing station on medicine ward B. Heating oven handles located in medicine ward A were contaminated with *C. difficile* on two separate visits. Molecular characterization of these *C. difficile* isolates revealed ribotypes 027 and 078.

### Patient isolates

Data pertaining to MRSA and *C. difficile* isolates from patients are presented in Tables 2 and 3, respectively. For MRSA, a total of 46 patient isolates were collected, representing nine different *spa* types, with *spa* type 2/t002 being the most prevalent. In one MRSA exposed room, the privacy curtain was contaminated with the same MRSA *spa* type that was identified in the patient, *spa* type 2/t002. Two patients were identified with MRSA *spa* type 1/t008 during the study period. However, only one of these patients was present in the ward when a chair located in the visiting room on the same ward as the patient was identified as being contaminated with MRSA *spa* type 1/t008. Although six patients were identified with MRSA *spa* type 437/t003, none of these patients were present in the surgical ward when a chair located in an unexposed room was found to be contaminated with *spa* type 437/t003. Despite nine different MRSA *spa* types identified in the patient population, six of these *spa* types were not detected on surfaces located in patient rooms or the ward environment.

For *C. difficile*, 22 patient specimens were collected during the study period; however, *C. difficile* could not be isolated from one patient specimen. For 21 *C. difficile* isolates, 11 ribotypes were identified, with ribotype 027 being the most prevalent in the patient population. During visit 4, ribotype 078 was identified from the majority of *C. difficile* contaminated surfaces located in patient rooms and the general environment. However, only one *C. difficile* patient was identified with ribotype 078 during the study period. Although this patient was admitted to the surgical ward shortly after visit 3, this patient was not present in the hospital during visit 4. Ribotype MOH-V was identified from one patient specimen; however, this patient was not present in the medicine or surgical wards when MOH-V was identified in an unexposed room and the ward environment. A total of seven *C. difficile* patients were identified with ribotype 027 during the study period. However none of these patients were admitted to the medicine ward where ribotype 027 was found in an unexposed room and the ward environment. A total of eight ribotypes identified from patient specimens were not recovered from the hospital environment.

## Discussion

To our knowledge, this is the first study to explore MRSA and *C. difficile* contamination, concurrently, in both patient rooms and the ward environment of a community hospital, longitudinally. The percentages of exposed patient rooms that were found to be contaminated with MRSA or *C. difficile* were lower compared to other studies [24-27]. This contrast in results may have been attributed to the number of patient rooms and surfaces investigated in addition to different sampling and culturing methodologies used among the studies.

Surfaces located in rooms with a *C. difficile* patient had increased odds of being contaminated with *C. difficile* compared to unexposed and MRSA exposed rooms, an unsurprising result given the potential for infected patients to contaminate their environment and the ability of *C. difficile* spores to survive in the hospital environment for months [8]. Interestingly, unexposed rooms were also identified as having a large number of surfaces contaminated with MRSA or *C. difficile*. Previous studies have also reported the presence of these HA pathogens on surfaces located in patient areas not occupied by MRSA or *C. difficile* patients [25,27,28]. Reasons for the extensive contamination of surfaces in unexposed patient rooms may include transfer of pathogens via hands or fomites of staff, patients, and visitors; misclassification of patient rooms due to unidentified colonization status of individuals; or failure of routine cleaning and disinfection practices. Although MRSA and *C. difficile* are susceptible to the disinfectants used in this facility, a quaternary ammonium compound and bleach, respectively, inadequate cleaning and disinfection practices cannot be excluded. In a multicentre study conducted by Carling and colleagues [29], only 49.5% (1748/3532) of high touch surfaces located in intensive care units were identified as being clean following terminal cleaning practices. Lastly, rooms housing known MRSA or *C. difficile*-infected individuals have enhanced cleaning protocols compared to other rooms, as a result, surfaces may not be cleaned as rigorously or as often in unexposed rooms. These data highlight the need for regular and proper cleaning of all hospital areas, not just those known to house patients with pathogens such as MRSA and *C. difficile*.

In patient rooms, the identification of MRSA or *C. difficile* on overbed tables, privacy curtains, and televisions is consistent with the literature [24,30-36], and presumably, any hand contact surface is at some degree of risk for contamination. While understanding higher risk sites is important, these data indicate the need for broad cleaning and disinfection, including surfaces that often receive less attention. In the present investigation, chairs and bulletin boards were sampled in patient rooms as it was noted that staff, patients, and visitors would move chairs by grasping the top surface of the chair back. Subsequently, MRSA and *C. difficile* were cultured from this surface with all positive chair backs specifically located in unexposed patient rooms. In unexposed and exposed patient rooms, bulletin boards were negative for MRSA but positive for *C. difficile*. Upon further investigation it was determined that in rooms with a patient under isolation precautions, tourniquets used for phlebotomies were secured to the bulletin boards. As *C. difficile* has been cultured from the skin of asymptomatic patients [37] and from the skin of patients with CDI [37,38], it is theorized that the tourniquets became contaminated with *C. difficile* which subsequently contaminated the bulletin board. Although results from the univariable analyses indicated that bulletin boards, compared to the end of the patient bed, were not significantly associated with *C. difficile* contamination, cork surfaces, compared to plastic and fabric surfaces, had significantly increased odds of being contaminated with *C. difficile*. In this investigation all bulletin boards in patient rooms were made of cork, a porous material with an irregular surface. Due to the surface characteristics of cork, which make this material difficult to clean and disinfect, and the likelihood that bulletin boards were rarely, if ever disinfected, it is possible that bulletin boards may have acted as a source of *C. difficile* dissemination in patient rooms. It is not clear why MRSA was not recovered from any of the bulletin boards in this investigation since phlebotomy tourniquets have also been documented as acting as a reservoir for MRSA [39].

Overall, 2.5% and 5.9% of surfaces in the ward environment were contaminated with MRSA or *C. difficile*, respectively. However, in the literature, the prevalence of MRSA and *C. difficile* contamination of the general hospital environment is extremely variable [4,28,40-44] and therefore difficult to compare results between studies. These differences in prevalence may be attributed to different study designs as well as the presence and number of MRSA and *C. difficile* patients during the study period.

In the general ward environment, surfaces covered in fabric, laminate, or plastic had the highest level of MRSA or *C. difficile* contamination; however there was no statistically significant association between type of material and contamination. As these materials can vary widely in their texture, they can pose a substantial problem with respect to cleaning and disinfection, and therefore act as potential sources of MRSA and *C. difficile* transmission and dissemination. *Staphylococcus aureus* is able to survive adverse environmental conditions and persist in the hospital environment [45] including surviving in hospital dust for nearly a year [7]. Experiments conducted using swatches of fabric and plastic have demonstrated that staphylococci can survive days to months after drying on these types of materials which are commonly found in the hospital environment [46]. In addition, *C. difficile* spores are highly resistant to environmental effects and many commonly used disinfectants [47,48], which can result in their survival for months to years in the hospital environment, unless physically removed or exposed to an adequate disinfectant [8].

In the ward environment, countertops, heating oven handles, computer keyboards, and chairs had the highest prevalence of MRSA or *C. difficile* contamination; however, there was no statistically significant association between specific surfaces and contamination with either pathogen. This lack of statistical association is in contrast to an earlier study investigating contamination in the ward environment of three hospitals in which chairs, hand rails, isolation carts, and sofas had significantly greater odds for MRSA contamination compared to computer keyboards [40]. However, in that particular study, the prevalence of MRSA contamination was considerably higher (11.8%) and the low prevalence of *C. difficile* contamination (2.4%) did not allow for statistical model construction. Nonetheless, results from the present investigation demonstrate that contaminated surfaces are common hand-touch sites that are frequently touched by staff and/or patients and visitors. Therefore, contamination may be attributed to lack of hand hygiene, hand hygiene practices that are ineffective at eliminating *C. difficile* spores, and/or inconsistent cleaning and disinfection protocols.

During visits 3–6, *C. difficile* was identified as the predominant pathogen contaminating the ward environment. Although MRSA patients were present in the medical and surgical wards for all six visits, *C. difficile* patients were only present in the wards for visits 4–6. For visit 4 (week 13), the prevalence of MRSA was relatively small despite an MRSA outbreak during weeks 8–13; however there was no significant association between the prevalence of MRSA contamination prior to, and following, the outbreak. As the MRSA outbreak occurred on a ward not involved in this investigation, the substantial decrease of MRSA in the ward environment may have been a result of overall increase in hand hygiene and restricting patient movement and visitors to prevent the outbreak spreading to other wards. Furthermore, an influenza outbreak, which also occurred in a ward not participating in this investigation, was declared during weeks 9–11 of the study period. Following this outbreak, the prevalence of *C. difficile* contamination in the ward environment of the medical and surgical wards fluctuated over the last three visits with a substantially higher prevalence observed on visit 4; however, there was no significant association between the prevalence of *C. difficile* contamination before and after the outbreak. This increase in *C. difficile* contamination in the environment during the latter part of this investigation may have been attributed to the presence of *C. difficile* patients in the study wards. However, research has demonstrated an association between the presence of the influenza virus and an increase in the incidence rate of CDI in hospitalised patients during the winter months [49,50]. Possible factors contributing to this association include antimicrobial use [49,50] and reductions in cleaning and disinfection of non-outbreak wards because of increased efforts directed towards outbreak wards. In the present investigation, the monthly CDI incidence rate increased from 0.73 to 0.83 cases/1,000 patient days following the influenza outbreak. It is possible that patients with *C. difficile* were not identified and isolated, or a focus on influenza outbreak management led to decreased infection control activities directed against *C. difficile,* subsequently resulting in an increase in *C. difficile* contamination of the general environment.

The predominance of *spa* types corresponding to CMRSA-2, a sequence type 5 (ST5) clone, also known as USA100, was not unexpected since it is the leading cause of HA-MRSA in Canada [1,51]. The distribution of MRSA strains in patient rooms, general ward areas, and patients was similar, albeit with more apparent strain diversity among patients than in the environment. It is unclear whether this indicates that CMRSA-2 is more adapted for surviving in the environment, as strain-dependent environmental persistence has not been investigated.

In total, 16 different toxigenic *C. difficile* ribotypes were identified. Ribotype 027 was predominant in the patient population and was the second most prevalent ribotype identified contaminating the ward environment. This specific ribotype is responsible for various outbreaks of CDI with increased severity, high relapse rates, and significant mortality [52,53]. Despite its association with outbreaks, this ribotype is also common in endemic disease and was the second most prevalent strain in a study of *C. difficile* isolates from Ontario diagnostic laboratories [54]. This is further evidence that while this strain is of particular concern, ribotype 027 can be present in patients and the environment in the absence of a CDI outbreak. Yet, overall, *C. difficile* typing data indicate less of a relationship between patient and environmental strains than with MRSA. In particular, ribotype 078 accounted for 64% of isolates from patient rooms and 36% from the ward environment but was identified in only one patient. Increases in the incidence of CDI caused by this particular strain have been reported internationally [55], including in Canada where 078 increased from 0.5% of CDI isolates in 2004/2005 to 1.6% in 2008 [56]. The reason for the high prevalence of 078 in the environment of this healthcare facility and the discordance between environmental and patient prevalence is not known. However, this particular ribotype is prevalent among *C. difficile* strains isolated from food animals in Canada [57-59] and was commonly identified in a study of Ontario household environments [60]. The participating hospital serves a rural community, which may increase the likelihood for exposure to 078 in the community, with subsequent transmission into the hospital. While ribotype 078 is referred to as a hypervirulent strain, it is not likely as pathogenic or transmissible as ribotype 027, based on fewer reports of severe disease or outbreaks associated with 078. Therefore, it is plausible that environmental contamination could occur more readily than patient disease if ribotype 078 is more common in people in the community but less able to cause disease, in comparison to ribotype 027. Given the lack of surveillance data involving non-diarrheic individuals, it is difficult to substantiate these hypotheses and more information is required regarding the epidemiology of this potentially emerging strain and the potential role of non-CDI patients, visitors, and staff in the contamination of the hospital environment.

The prevalence of toxinotype variants (toxinotypes other than toxinotype 0) in patients was striking, with only 14% of isolates belonging to toxinotype 0. The high prevalence and diversity of toxinotype variants seen in our study hospital have not been reported previously in a patient population and is in contrast with an earlier multicentre study in the province where ribotype 001, a toxinotype 0 strain, was most common (25.5%) [54]. Similarly, the high prevalence of patient strains possessing the binary toxin strain (81%) was remarkably high. This toxin is most commonly found in hypervirulent strains, although it is not clear whether it is an important virulence factor or simply just commonly present in strains that are more virulent.

This study has several limitations. For *C. difficile*, several ribotypes present in the hospital environment were not identified from patient specimens. This discordance may have been attributed to isolates not being collected or patients with *C. difficile* not being identified because of a lack of routine screening or the use of a diagnostic test with moderate sensitivity [61,62]. In addition, not all surfaces were sampled each visit since some surfaces were cleaned just prior to our arrival for sampling. Lastly, the number of MRSA and *C. difficile* exposed rooms available for sampling was limited, which may have resulted in a lack of power to identify significant risk factors associated with surfaces being contaminated with MRSA or *C. difficile*.

## Conclusions

In conclusion, the results from this investigation offer detailed information on MRSA and *C. difficile* contamination in the hospital environment. The identification of specific surfaces, materials, and locations as having an increased prevalence of MRSA and *C. difficile* contamination can be used by hospital personnel for surveillance purposes to assess and implement effective control measures for reducing the transmission of MRSA and *C. difficile* within the healthcare setting. In addition, this study demonstrates the variety of MRSA and *C. difficile* strains that can be found in patient populations and the hospital environment. Further research investigating the persistence of specific MRSA and *C. difficile* strains in the hospital environment may be warranted.

## Abbreviations

MRSA: Meticillin-resistant *Staphylococcus aureus*; CMRSA: Canadian epidemic meticillin-resistant *Staphylococcus aureus*; CDI: *Clostridium difficile* infection; HA: Healthcare-associated; CA: Community-associated; PVL: Panton-Valentine leukocidin; tcdA: Toxin A; tcdB: Toxin B; cdtA: Binary toxin; ST: Sequence type; OR: Odds ratio; N: Number.

## Competing interests

The authors declare that they have no competing interests.

## Authors’ contributions

MCF contributed to study design, data collection, analysis, and drafting of the manuscript. DLP and OB contributed to study design and statistical analysis. JSW contributed to study design and molecular analysis. RRS contributed to study design. All authors contributed to the editing and final version of the manuscript.

## Pre-publication history

The pre-publication history for this paper can be accessed here:

http://www.biomedcentral.com/1471-2334/13/342/prepub
